# Two-stage revision arthroplasty for *Mycobacterium Tuberculosis* periprosthetic joint infection: An outcome analysis

**DOI:** 10.1371/journal.pone.0203585

**Published:** 2018-09-07

**Authors:** Chih-Hsiang Chang, Chih-Chien Hu, Yuhan Chang, Pang-Hsin Hsieh, Hsin-Nung Shih, Steve Wen-Neng Ueng

**Affiliations:** 1 Bone and Joint Research Center, Chang Gung Memorial Hospital, Linkou, Taiwan; 2 Department of Orthopedic Surgery, Chang Gung Memorial Hospital, Linkou, Taiwan; 3 Graduate Institute of Clinical Medical Sciences, College of Medicine, Chang Gung University, Taoyuan, Taiwan; 4 College of Medicine, Chang Gung University, Taoyuan, Taiwan; INMI Spallanzani, ITALY

## Abstract

**Background:**

*Mycobacterium tuberculosis* periprosthetic joint infection (TBPJI) is a rare complication of hip/knee joint arthroplasty. The outcomes of hip/knee TBPJI treatment are still unreported. The objective of this study was to investigate the outcomes of hip/knee TBPJI following treatment with two-stage exchange arthroplasty.

**Materials and methods:**

From 2003 to 2013, 11 patients with TBPJI (six hips and five knees) were treated with two-stage exchange arthroplasty at our institution. We collected and analyzed variables including demographic data, comorbidities, microbiological data, duration of symptoms, and types of antibiotic used in bone cement.

**Results:**

At the most recent follow-up, the success rate of two-stage exchange arthroplasty was 63.3% (7 of 11). All five knee treatments resulted in infection eradication and successful prosthesis reimplantation. However, only two hip TBPJI treatments resulted in successful outcomes; two patients died and two experienced chronic infection. Overall, secondary bacterial infections were common in patients with TBPJI (5 of 11 cases, 45.5%). Streptomycin in bone cement increased the success rate (83.33% *vs*. 40%).

**Conclusion:**

More than one third of the patients treated with two-stage exchange arthroplasty for TBPJI showed infection relapse or uncontrolled infection. Streptomycin-loaded interim cement spacers appeared to help ensure successful treatment. Routine *M*. *tuberculosis* culture is recommended when treating TBPJI in areas of high tuberculosis prevalence.

## Introduction

Total joint arthroplasty is a useful procedure that provides significant pain relief and improves activities of daily living with durable survivorship[1, 2]. However, periprosthetic joint infection (PJI) is one of the most devastating complications following joint arthroplasty. PJI accounts for 14.8% of revisions performed after hip arthroplasty and is the most common cause of revision after knee arthroplasty (25.2%)[3, 4]. The most common pathogens involved are *Staphylococcus* species, which constitute 50–60% of all isolates[5–7]. In patients infected with common gram-positive or -negative bacteria, two-stage revision with interim antibiotic-impregnated polymethyl methacrylate spacer implantation provides a success rate approaching 90% and is the current gold-standard for controlling chronic PJI[5–10].

*Mycobacterium tuberculosis* PJI (TBPJI) is a rare complication that accounts for <1% of PJI cases[11]. The identification of TBPJI is often complicated by a lack of clinical suspicion, varied presentations, and the presence of concomitant secondary bacterial infections. Patients with unsuspected tuberculotic, septic arthritis discovered at the time of implantation or in the early postoperative period can be successfully treated with anti-*M*. *tuberculosis* drugs for 12–18 months[12, 13]. However, there are only a few case reports of the successful treatment of chronic TBPJI, and the optimal therapy remains unclear[11, 14–28].

To the best of our knowledge, no reports have assessed the success rate of prosthesis reimplantation after TBPJI. The purpose of this study was to answer the following questions: (1) what is the success rate in terms of infection eradication following two-stage exchange arthroplasty in patients with hip/knee TBPJI; and (2) what patient-, infection-, and treatment-related variables are associated with treatment success or failure?

## Materials and methods

This study was approved by the Institutional Review Board of Chang Gung Memorial Hospital (No. 102-1846B). We retrospectively reviewed our institutional joint arthroplasty database to identify patients who were diagnosed with a hip or knee TBPJI at our institution between January 2003 and December 2013. TBPJI was defined as *M*. *tuberculosis*-positive cultures from two or more joint aspiration or surgical samples. *M*. *tuberculosis* was cultured based on the method previously reported by Levin *et al*. in 1950[29] and involved incubation for up to 12 weeks. A concomitant infection was defined as simultaneous positive bacterial cultures from two or more joint aspiration or surgical samples. All PJIs were late, chronic infections that lasted ≥3 weeks[30].

We identified 342 patients with hip/knee PJIs (hip: 180, knee: 162) at our institution during the study period (Table 1). Thirteen (3.8%, 13/342) hips/knees were diagnosed with TBPJI. We reviewed patient age, sex, comorbidities, microbiological study results, antimicrobial agents used, and final outcomes in all patients with hip/knee TBPJI. All patients except two with chronic hip/knee TBPJI were treated with resection arthroplasty for infection control; one patient with hip TBPJI was extremely elderly (101 years) and refused to undergo resection arthroplasty, whereas the other patient, an 80-year-old with knee TBPJI, had poorly controlled Parkinson’s and pulmonary tuberculosis and refused to undergo resection arthroplasty for infection eradication. Finally, 11 patients, representing five knees and six hips, were treated with two-stage exchange arthroplasty and included in the outcome analysis. In brief, resection arthroplasty for PJIs included radical debridement, removal of the prosthesis, antibiotic-loaded bone cement implantation, and administration of systemic antimicrobial agents for infection control.

**Table 1:** Demographics of hip and knee prosthetic joint infections.

Demographic characteristics			Hip (n = 180)	%	Knee (n = 162)	%
	Sex					
	Male		126	70	87	54
	Female		54	30	75	46
**Aerobic**	214		109		105	
**Anaerobes**	12		12		0	
**Fungus**	10		6		4	
**Mycobacterium**	13		6		7	
**Negative culture**	93		47		46	

Delayed reimplantation of the prosthesis was performed after successful antimicrobial therapy as defined by the absence of signs of infection, including: no resting pain, local heat, erythema, or discharging sinus and both erythrocyte sedimentation and serum C-reactive protein levels returning to normal. If the infection status remained positive, another exchange arthroplasty with a cement spacer was performed. Permanent excision arthroplasty at the hip or fusion at the knee was considered if resection arthroplasty for infection control failed twice.

All patients were treated with oral multidrug anti-*M*. *tuberculosis* medications according to the clinicians' discretion during follow-up, and antibiotic-loaded bone cement spacers were used in every patient who underwent resection arthroplasty. All patients received a minimum of 10 months of anti-*M*. *tuberculosis* medication before reimplantation of the prosthesis. Anti-*M*. *tuberculosis* medication was continued beyond 12 months (a full course of treatment) even after reimplantation of the prosthesis. Patients were routinely checked for liver function and visual ability during follow-up.

Treatment success was defined as a well-functioning joint without relapse of tuberculosis or other infections after prosthesis reimplantation during follow-up. Treatment failure was defined as any of the following after reimplantation: (1) PJI attributable to the presence of the original microorganism (infection relapse) or a different strain (reinfection); (2) development of a sinus tract infection; or (3) death related to the PJI.

We performed statistical analyses using SPSS software (Version 22.0; SPSS Inc., Chicago, IL, USA). The Mann-Whitney U-test was used to analyze continuous variables and Fisher’s exact test was to used analyze dichotomous variables. Statistical significance was set at a p-value of <0.05.

## Results

During this 11-year study, 3.8% (13/342) of PJI cases involved TBPJI. Five men and eight women were included in the study. Seven affected hips and six knees were assessed. The average patient age was 74 years (range: 48–101 years; Table 2). The average time between the date of index surgery (defined as previous hip or knee replacement surgery) and symptom onset was 12 months (range: 3–29 months). The average duration of symptoms was 2 months (range: 0.5–6 months) and the average time between the diagnosis of PJI and confirmation of TBPJI diagnosis was 5 months (range: 1.5–16 months). The minimum follow-up period for patients with TBPJI was 15 months (mean: 63.4 months; range: 15–133 months). At the final follow-up, all five cases of knee TBPJI resulted in infection eradication and successful prosthesis reimplantation. However, only two of six hip TBPJI cases resulted in successful outcomes. Two hip TBPJI patients died, one from liver cirrhosis related to uncontrolled sepsis and the other from medical complications related to chronic renal insufficiency under regular hemodialysis. Another two patients developed chronic discharging sinus after refusing to undergo an additional operation or receive anti-*M*. *tuberculosis* medication and recurrent infection status after permanent resection arthroplasty (Table 2). The overall success rate for the treatment of hip/knee TBPJI using two-stage exchange arthroplasty was 63.6% (7 of 11 cases). Despite two patient deaths and one patient who refused to receive a further operation and anti-*M*. *tuberculosis* medication, all other patients underwent surgical debridement two to four times. Both patients (patients 12 and 13) who refused to undergo resection arthroplasty developed chronic infections.

**Table 2:** Summary of patients’ clinical characteristics in tuberculosis mycobacterial periprosthetic joint infection(TB PJI).

No	Age/ Sex/ Site	Microbe	Comorbidity	Antibiotic in cement spacer	ATT therapy	Time between the date of index surgery until symptoms	Duration of symptoms	Time between infection till diagnosis	Number of debridement Surgeries	Operative procedure	Outcome	F/U
1	74/F/H	MRSA+TB	HTN	Vanco+Pipril	INH, RIF, PZA, EMB(12)	16m	1m	16m	2	Two stage	Infection cleared	133m
2	77/F/H	TB	HTN, DM, Pulmonary TB	Strep+ vanco	INH, RIF, PZA, EMB(15)	3m	1m	3m	3	Two stage	Infection cleared	61m
3	84/M/H	CoNS + TB	non	Vanco		5m	0.5m	3m	1	Two stage	Chronic infection	78m
4	57/F/H	CoNS + TB	DM, HTN	Vanco+ strep	INH, RIF, PZA, EMB(12)	3m	0.5m	9m	4	Two stage	Resection arthroplasty	85m
5	48/M/H	MSSA+TB	Cirrhosis, alcoholism	Teico+Ceft		24m	1m	3m	1	Two stage	Dead	
6	82/F/H	TB	DM, HTN, CVA, CKD	Vanco+Azt		12m	0.5m	1.5 m	1	Two stage	Dead	
7	73/F/K	CoNS + TB	HTN	Vanco+ Strep	INH, RIF, PZA(15)	29m	1m	3m	2	Two stage	Infection cleared	111m
8	72/F/K	TB	DM	Vanco+Ceft	INH, RIF, PZA, EMB(14)	24m	4m	1.5 m	2	Two stage	Infection cleared	34m
9	72/M/K	TB	Parkinsonism, Prostate Ca, Pituitary adenoma, pulmonary TB	Strep	INH, RIF, PZA(12)	7m	2m	2.5 m	2	Two stage	Infection cleared	24m
10	70/F/K	TB	HTN	Strep	INH, RIF, PZA, EMB(8)/ INH, RIF(4)	4m	4m	5 m	3	Two stage	Infection cleared	27m
11	81/M/K	TB	HTN, HCV, Thyroid Ca, Prostate Ca	Strep	INH, RIF, PZA, EMB(14)	8m	6m	8 m	2	Two stage	Infection cleared	27m
12	101/F/H	CoNS + TB	non		INH, RIF, PZA, EMB	20m	2m	6m	1	DAIR	Chronic infection	
13	80/M/K	TB	Pulmonary TB, parkinsonism		EMB, TBN, PAS, CS, STREP	11m	1m	3 m	1	DAIR	Chronic infection	

Note. Abbreviation

MRSA: Methicillin resistant staphylococcus aureus

MSSA: Methicillin sensitive staphylococcus aureus

CoNS: Coagulation-negative staphylococcus

HTN: Hypertension

DM: Diabetes mellitus

CVA: Cerebrovascular accident

Vanco: Vancomycin

Strep: Streptomycin

Pipril: Piperacillin

Teico: Teicoplanin

Ceft: Ceftazidime

Azt: Azreonam

ATT treatment: Anti-TB treatment

RIF: Rifampicin

INH: Isoniazid

PZA: Pyrazinamide

EMB: Ethambutol

PAS: para-aminosalicylic acid

TBN: prothionamide

CS: cycloserine

Two stage: Two-stage exchange arthroplasty

DAIR: Debridement, antibiotics, and implant retention surgery

The success rate for the treatment of knee joints was better than that for hip joints (100% *vs*. 33.3%, 5/5 *vs*. 2/6). If we excluded mortality related to medical complications and chronic infections due to incomplete treatment, the success rate for hip infections was adjusted to 50% (2 of 4 hips). Several potential differences in relative success rates were noted: 1) those between male and female patients (66.7 *vs*. 83.3%), 2) those between patients receiving and those not receiving treatment with a streptomycin-impregnated spacer (83.33% *vs*. 66.7%), 3) those stemming from concomitant presence of *M*. *tuberculosis* in both the joint and lung (71.4% *vs*. 100%), and 4) those related to the presence or absence of concomitant infection (50% *vs*. 100%) (Table 3).

**Table 3:** Comparison between success and failure patients in tuberculosis mycobacterial periprosthetic joint infection(TBPJI).

	Success (n = 7)	Failure (n = 2)	Success rate %
Male	2	1	66.7 83.3
Female	5	1	
Streptomycin in spacer	5	1	83.3 66.7
Streptomycin not in spacer	2	1	
Hip	2	2	50
Knee	5	0	100
No pulmonary TB	5	2	71.4
Pulmonary TB	2	0	100
Concomitant infection	2	2	50
No concomitant infection	5	0	100
Age, average years (range)	74.1 y/o (70–81)	66y/o (48–84)	

Note. Exclude the dead related to medical complication and chronic infection due to refusing further operation and anti-TB medication

Two patients died: patient No. 5 had severe liver cirrhosis (Child-Pugh score C) and died from uncontrolled sepsis, whereas patient No. 6 developed end-stage renal disease under regular hemodialysis and died due to medical complications. Some patients developed chronic infections: patient No. 3 experienced an unexpected positive *M*. *tuberculosis* culture after replantation and refused to received further anti-*M*. *tuberculosis* medications or surgical interventions. Patient No. 4 was initially treated for pyogenic PJI and received serial debridement for the uncontrolled infection. The presence of *M*. *tuberculosis* was not identified until 9 months after symptom onset. However, due to persistent wound discharge and uncontrolled infection even following streptomycin-loaded cement implantation and anti-*M*. *tuberculosis* medication, the patient underwent permanent resection arthroplasty.

In all cases, *M*. *tuberculosis* was sensitive to rifampicin, isoniazid, and pyrazinamide. There were five patients with concomitant infections (5 of 11; 45.5%, Table 2). The most common coinfection was with coagulation-negative *Staphylococcus* (three patients), followed by methicillin-resistant *Staphylococcus aureus* and methicillin-sensitive *Staphylococcus aureus* (one patient each). All patients with concomitant *Staphylococcus* species infections were treated with vancomycin-loaded bone cement. No further parenteral antibiotics were prescribed for concomitant infections. Patients with concomitant infections had a lower success rate than those without (50% *vs*. 100%, Table 3).

## Discussion

PJI is one of the most serious complications following joint arthroplasty. TBPJI is rare, accounting for <1% of PJI cases[11]. The presentation of tuberculosis varies and the infection can even be misdiagnosed as an aseptic condition. Because TBPJI is uncommon, it does not readily elicit clinical suspicion, and the presence of concomitant bacterial infections can direct treatment entirely toward non-*M*. *tuberculosis* pathogens. Failure to perform mycobacterial culture intra-operatively can delay diagnosis. The overall success rate of the treatment of hip/knee TBPJI with two-stage exchange arthroplasty was 63.6% (7 of 11 patients) in this study. The success rate for knees was better than that for hips (100% *vs*. 33.3%, 5/5 *vs*. 2/6). If mortality related to medical complications and chronic infection due to incomplete treatment was excluded, the success rate for hip infections was adjusted to 50% (2/4). Several potential factors affecting relative success rates were sex, use of a streptomycin-impregnated spacer, concomitant pulmonary *M*. *tuberculosis* infection, and concomitant bacterial infection (Table 3).

*M*. *tuberculosis* infections may occur in native joints[31–33] or as periprosthetic infections. The latter may persist in a chronic state[20, 22, 23, 25, 26, 34, 35], reemerge upon the reactivation of previous *M*. *tuberculosis* arthritis[16, 17, 36], or persist as misdiagnosed arthritis[18, 24] (Table 4). *M*. *tuberculosis* infection in native joints can be treated successfully with antibiotics with or without surgical debridement. When dealing with mycobacterial arthritis, treatment using surgical debridement and anti-*M*. *tuberculosis* medication is followed by arthroplasty when the knee and hip joints are damaged[37, 38]. Primary *M*. *tuberculosis* arthritis may be reactivated years after arthroplasty and ostensible infection eradication. Such reactivated infections can be treated successfully with debridement surgery or two-stage exchange arthroplasty[16, 17, 36]. Recurrent or acute (<3 weeks of symptoms) *M*. *tuberculosis* infections can be successfully treated with antibiotics for 12–18 months[12, 13]. Debridement surgery[17, 20, 22, 24, 36], arthrodesis[14, 26], or two-stage exchange arthroplasty[15, 16, 18, 22, 25, 34, 35] followed by prolonged anti-*M*. *tuberculosis* medication have been reported for the treatment of TBPJI. However, only case reports have been reported in the literature, and the optimal therapeutic strategies remain unclear. With this in mind, this report represents the results of a retrospective analysis, which aimed to characterize the success rate of *M*. *tuberculosis* infection elimination in the context of two-stage exchange arthroplasty for knee or hip TBPJI. We also sought to characterize differences between TBPJI and pyogenic PJI.

**Table 4:** Treatment outcomes of cases reported in the literature.

Study	P’t age, y/gender/joint	Microbe	ATT treatment	Diagnosis of infection after arthroplasty	Operative procedure	Infection type
Lee[20]	79/F/K	TB	INH, RIF, EMB(12)	2m	DAIR	Chronic
Spinner[39]	70/F/K	CoNS + TB	RIF, EMB(12)	60m	Two stage	Chronic
Shanbhag[25]	59/F/H	TB	RIF, EMB, PZA(12)	15m	DAIR	Miss-diagnosed TB arthritis
Neogi[37]	73/F/K	TB	INH, RIF, PZA, EMB(18)	168m	Medication only	Chronic
Harwin[16]	60/F/K	TB	INH, RIF, PZA,EMB(12); RIF, INH(9)	24m	Two stage	Reactivation
Tokumoto[26]	70/F/K 72/F/H	TB	INH, EMB (12)	456m/20m	Arthrodesis	Chronic
Wolfgang[35]	61/M/K	TB	INH, RIF (24)	12m	Two stage	Chronic
Ueng[34]	62/M/H 63/M/H	TB	INH, RIF, EMB (24, 12)	18m/168m	Two stage/ resection arthroplasty	Chronic
Marmor[22]	65/F/K 66/M/K 77/F/K	TB	INH, RIF, PZA (6, 6); INH, EMB, PZA(8)	3m/ 2m/4m	Two stage/two stage/ DAIR	Chronic
Kreder[19]	66/F/H	TB	INH, EMB, PZA (9)	48m	acetabulum revision	Chronic
Carrega[15]	72/F/K 79/F/H 92/F/H 80/F/K	TB/ TB/ CoNS + TB/ TB	INH, RIF, EMB (2)/INH, RIF (10)/ INH, RIF, EMB(9)/ INH, RIF, EMB(9)/ INH, RIF, EMB (2); INH, RIF (12)	84m/12m/36m/12m	Two stage/ Two stage/ One stage/ Two stage	Chronic
Klein[18]	36/F/K	TB	INH, RIF, PZA, EMB (20)	12m	Two stage	Miss-diagnosed TB arthritis
Al-Shaikh[14]	73/F/K	CoNS + TB	INH, RIF, PZA (12); INH, RIF, EMB (9)	8m	Arthrodesis	Chronic
Hugate[17]	71/M/H	TB	INH, RIF, EMB (12)	5m	DAIR	Reactivation
Khater[36]	75/F/K	TB	INH, RIF, PZA, EMB (unknown)	3m	DAIR	Reactivation
Delrieu[27]	75/F/H 69/F/H	TB/TB	Unknown Unknown	10y/1y	Two stage Two stage	Chronic Chronic
Kaya [28]	72/F/H	MRSA + TB	INH, EMB, PZA (unknown)	9y	Resection arthroplasty	Chronic

Note.

K: Knee

H: Hip

CoNS: Coagulation-negative staphylococcus

MRSA: Methicillin resistant staphylococcus aureus

ATT treatment: Anti-TB treatment

RIF: Rifampicin

INH: Isoniazid

PZA: Pyrazinamide

EMB: Ethambutol

Two stage: Two-stage exchange arthroplasty

DAIR: Debridement, antibiotics, and implant retention surgery

Average age(range): 71.23 y/o, 36~92 y/o

Hip/Knee: 11/14

Male/Female: 5/20

TBPJI is a rare complication following total joint arthroplasty. The incidence of TBPJI among all PJI cases over a 22-year period was 0.3% (7/2,116) in one study[11]. Our data from PJI cases treated over an 11-year period showed that 3.8% (13/342) of PJI cases involved TBPJI. The 2015 annual report from the World Health Organization identified Southeast Asia as having a high prevalence of tuberculosis compared to the global average. The incidence of TBPJI found in our analysis is much higher than that reported in earlier studies. This is likely explained by the high prevalence of tuberculosis in our country[32, 33]. As we work in an area of high tuberculosis prevalence, we routinely perform *M*. *tuberculosis* culture during PJI debridement. After analyzing the demographic data, TBPJI patients were older (74 years, 48–101 year) than PJI patients in general[5–10]. In our literature review (Table 4), the average age of TBPJI patients was 71.23 years, which was similar to that identified in our patient series (mean age: 74 years). After analyzing the sex of our patients, women were more common (eight compared to five men), which may reflect the sex distribution among the elderly and the predominance of female patients in the original joint replacement population.

The average duration of symptoms was 2 months (range: 2 weeks to 6 months). This is consistent with the ambiguity of tuberculosis symptoms, which were sometimes so minor that patients took considerable time before seeking help. Diagnoses were delayed by 6 weeks to 16 months after symptom onset, with *M*. *tuberculosis* culture often conducted following treatment failure for more general bacterial infections. The diagnosis of TBPJI was commonly delayed due to a lack of clinical suspicion. Uncontrolled infections due to failure to recognize and treat tuberculosis commonly resulted in repeated surgical debridement or resection arthroplasty. When diagnosing TBPJI, histological assessment supports the suspicion of *M*. *tuberculosis* infection, while the final diagnosis relies on culture. Traditional *M*. *tuberculosis* culturing is very time consuming, requiring 6–12 weeks. Improvements in culture efficacy have been recommended[39]. We currently use the recently developed polymerase chain reaction for the detection of *M*. *tuberculosis* (TBPCR), which provides highly sensitive *M*. *tuberculosis* identification in just a few hours[40]. The near-immediate diagnosis provided by this method greatly shortens delays before treatment initiation. Theoretically, PCR cannot distinguish between the presence of ongoing or previous *M*. *tuberculosis* infections, which may lead to false positive results in patients with a previous case of pulmonary tuberculosis. However, in this study, TBPCR was correlated with infection control in cases of second-stage reimplantation. Furthermore, traditional PCR does not identify a strain’s sensitivity to antibiotics. However, new technologies, such as GeneXper, utilize genotyping methods to rapidly identify resistant strains. At our hospital, we used the Roche COBAS TaqMan assay, which relies on traditional *M*. *tuberculosis* culture to determine antibiotic sensitivity.

*M*. *tuberculosis* is an extremely slow-growing pathogen, requiring 6–12 weeks for the completion of laboratory diagnostic cultures[41]. This slow growth complicates diagnosis and treatment. While the results of TBPCR prompt clinicians to use anti-*M*. *tuberculosis* medications, there is no consensus on the optimal duration of treatment. Considering that TBPJI can emerge from the reactivation of a latent disease, prolonged multi-drug therapy for a minimum of 6–9 months was our guiding treatment principle. All patients reported in this study received prolonged anti-*M*. *tuberculosis* multidrug therapy for 12–15 months. We recommend therapy for TBPJI for at least 10 months before reimplantation. The success rate could be increase if streptomycin is impregnated in the cement spacer.

Two-stage exchange arthroplasty has been the gold-standard procedure for treating PJI[5–10]. Because two-stage exchange arthroplasty is the gold-standard worldwide and, in our experience, poor infection control is obtained using debridement surgery, we prefer the former method at our hospital. It is possible to debride the entire joint radically after removing the prosthesis. However, treatment was successful in only 7 of 11 TBPJI patients using this strategy (63.6%). Results were worse for hips than for knees (2/6, or 33.33%). Two patients died, one due to a chronically infected discharging sinus and the other after undergoing a permanent Girdlestone procedure due to uncontrolled infection after repeated debridement surgery. Both patients who died were in a poor medical condition. One had liver cirrhosis with a Child-Pugh score of C and the other had chronic renal insufficiency and was receiving regular hemodialysis. The cirrhotic patient died from hip PJI-related sepsis, whereas the dialysis patient died from medical complications 1.25 years after experiencing TBPJI. A previous study reported that patients with liver cirrhosis who develop a PJI experience a higher rate of infection recurrence after two-stage exchange arthroplasty[42], possibly due to poor immune status. Although two patients died due to underlying disease, including one who refused further resection arthroplasty, only one patient needed to undergo permanent resection arthroplasty (patient No. 4). The success rate was 66.67% (2/3).

Regarding the treatment of arthritis associated with *M*. *tuberculosis*, joint destruction from unsuspected tuberculosis that was initially treated with arthroplasty can be treated successfully with anti-*M*. *tuberculosis* medication without further surgical intervention[12, 13]. However, should antibiotic treatment fail, surgical debridement or two-stage exchange arthroplasty followed by prolong anti-*M*. *tuberculosis* medication may be required[18, 24]. The results of knee PJI treatment were much better than those for hip PJI treatment (100% success rate [5/5]). However, this might be a coincidence, because apart from the patients who died or refused further management, there was only one patient who failed to respond and underwent permanent resection arthroplasty. When treating knee TBPJI, symptoms are easily detected and culture materials can be easily recovered via knee aspiration. Streptomycin-impregnated cement spacers were used in most cases of knee TBPJI (4/5, or 80%). This may have further improved the treatment success rate.

Concomitant infection is common in patients with TBPJI and could interfere with pathogen assessment. The rate of concomitant infection varies from 40% to 100% in the literature[11, 15, 19]. In the present study, 45.5% (5/11) of patients had concomitant infections. *Staphylococcus* species were the most common pathogens. Given that concomitant infections may direct focus away from *M*. *tuberculosis* due to its prolonged culture requirements[41], assessment for atypical microorganisms should always be considered[21]. While a new definition of PJI was published in 2011[43], the types of cultures to perform were not addressed. For patients living in areas with a high prevalence of *M*. *tuberculosis* infection, routine *M*. *tuberculosis* culture is recommended during the 1^st^ stage of PJI treatment based on the relatively high incidence of TBPJI reported in this study. About one fifth to one third of patients who have tuberculosis involving a bone or joint have a history of pulmonary disease[20, 44, 45]. In this study, three TBPJI cases had concomitant pulmonary tuberculosis, indicating that such concomitant infections should raise suspicions of TBPJI.

Surgical treatment for TBPJI is controversial. The successful treatment of TBPJI has been achieved by debridement surgery combined with prolonged anti-*M*. *tuberculosis* medication[17, 20, 22, 24, 36]. However, some studies have suggested two-stage exchange arthroplasty for improving results[11, 21, 34, 35]. Some reports have been published on two-stage exchange arthroplasty as the gold-standard for treating TBPJI[15, 16, 18, 22, 25, 26, 34, 35]. In the present study, surgical debridement combined with prosthesis retention and prolonged anti-*M*. *tuberculosis* medication was unable to eradicate the infection (0/2, 0%). Two-stage exchange arthroplasty yielded a higher success rate (7/11, 63.6%).

The application of antibiotic-loaded bone cement (ALBC) spacers to manage patients between disease stages has recently become a common approach[5–10]. Streptomycin-loaded bone cement is recognized as an effective treatment for tuberculous osteomyelitis[46]. High therapeutic levels can be reached in local synovial fluid with relatively safe serum levels after local implantation[46]. In this study, the use of streptomycin-loaded bone cement resulted in highly successful TBPJI treatments (5/6, or 83.3%). However, due to the small sample size, the benefit of this approach should be further examined (83.3% *vs*. 66.7%, Table 3). If streptomycin is not available, isoniazid is another choice when loading ALBC for the treatment of TBPJI[47]. Drug resistance has been a concern when using bone cement impregnated with antibiotics. However, we found no difference in drug resistance between patients treated with ALBC and those treated without ALBC in cases of PJI and TBPJI.

The risk factors for TBPJI have been reported as old age, systemic or intra-articular steroid administration, immunosuppression, and surgical trauma[15, 16, 20, 48]. In this study, patients were older than PJI patients in general (74 years, 48–101 years). However, we did not observe more immunosuppression or steroid administration. The etiology of TBPJI has been summarized as the hematogenous spread or reactivation of latent disease[20]. Unsuspected tuberculosis in septic arthritis treated by implantation or diagnosis in the early postoperative period has been reported[12, 13, 18, 24]. However, all cases in this study showed no such joint destruction patterns. Instead, all cases involved common degenerative osteoarthritis treated with arthroplasty. In all patients, we believe that TBPJI was caused by the reactivation of latent disease, potentially of pulmonary origin. This was supported by the fact that three patients (3/13, or 23%) had concomitant pulmonary tuberculosis.

This study had some limitations. The most serious of these were the small sample size and the fact that this was a retrospective review performed over an extended period of time. The latter resulted in variability in several factors, including operating surgeons, type of antibiotic used in bone cement, duration and choice of anti-*M*. *tubcerulosis* medications, and interval between resection arthroplasty and reimplantation. The rarity of TBPJI made it impossible to perform a randomized clinical trial. Despite these limitations, we believe that our findings provide rare outcome data concerning the use of two-stage exchange arthroplasty for the treatment of TBPJI.

In conclusion, TBPJI is a rare condition that was found in only 3.8% (13/342) of PJI patients in this study. The overall results of TBPJI treatment with exchange arthroplasty were not satisfactory (63.3% success rate [7/11]). If we excluded death that was not related to treatment and patients who refused further management, the success rate increased to 77.8% (7/9) even after repeated surgical debridement. If streptomycin was impregnated in the cement spacer, the success rate increased to 83.3% (5/6). Routine *M*. *tuberculosis* culture is recommended when treating PJI cases in areas of high tuberculosis prevalence, and the application of TBPCR can greatly shorten the diagnosis time to a few hours. However, even with exchange arthroplasty and prolonged multi-drug anti-*M*. *tuberculosis* medication, the overall results of this treatment approach were not satisfactory.
